# Evenly Distributed Protein Intake over 3 Meals Augments Resistance Exercise–Induced Muscle Hypertrophy in Healthy Young Men

**DOI:** 10.1093/jn/nxaa101

**Published:** 2020-04-22

**Authors:** Jun Yasuda, Toshiki Tomita, Takuma Arimitsu, Satoshi Fujita

**Affiliations:** Faculty of Sport and Health Science, Ritsumeikan University, Nojihigashi, Kusatsu, Shiga, Japan; Faculty of Sport and Health Science, Ritsumeikan University, Nojihigashi, Kusatsu, Shiga, Japan; Faculty of Sport and Health Science, Ritsumeikan University, Nojihigashi, Kusatsu, Shiga, Japan; Faculty of Sport and Health Science, Ritsumeikan University, Nojihigashi, Kusatsu, Shiga, Japan

**Keywords:** muscle hypertrophy, protein intake, protein distribution, resistance training, young subjects

## Abstract

**Background:**

Although daily protein intake (PI) has been reported to be essential for regulating muscle mass, the distribution of daily PI in individuals is typically the lowest at breakfast and skewed toward dinner. Skewed protein intake patterns and inadequate PI at breakfast were reported to be negative factors for muscle maintenance.

**Objectives:**

This study examined whether a protein-enriched meal at breakfast is more effective for muscle accretion compared with the typical skewed PI pattern.

**Methods:**

This 12-wk, parallel-group, randomized clinical trial included 26 men (means ± SEs; age: 20.8 ± 0.4 y; BMI: 21.8 ± 0.4 kg/m^2^). The “high breakfast” (HBR) group (*n* = 12) consumed a protein-enriched meal at breakfast providing a PI of 0.33 g/kg body weight (BW); their PI at lunch (0.46 g/kg BW) and dinner (0.48 g/kg BW) provided an adequate overall daily PI (1.30 g/kg BW/d). The “low breakfast” (LBR) group (*n* = 14) consumed 0.12 g protein/kg BW at breakfast; intakes at lunch (0.45 g/kg BW) and dinner (0.83 g/kg BW) yielded the same daily PI as in the HBR group. The participants performed supervised resistance training (RT) 3 times per week (75–80% 1-repetition maximum; 3 sets × 10 repetitions). DXA was used to measure the primary outcome variable, that is, total lean soft tissue mass (LTM).

**Results:**

The total LTM at baseline did not differ between the HBR (52.4 ± 1.3 kg) and LBR (53.4 ± 1.2 kg) groups. After the intervention, increases in total LTM were significant in both groups, with that in the HBR group (2.5 ± 0.3 kg) tending to be greater than that in the LBR group (1.8 ± 0.3 kg) (*P* = 0.06), with a large effect size (Cohen *d *= 0.795).

**Conclusions:**

For RT-induced muscle hypertrophy in healthy young men, consuming a protein-enriched meal at breakfast and less protein at dinner while achieving an adequate overall PI is more effective than consuming more protein at dinner.

This study was registered at University hospital Medical Information Network (UMIN) Clinical Trials Registry as UMIN000037583 (https://upload.umin.ac.jp/cgi-open-bin/ctr_e/ctr_view.cgi?recptno=R000042763).

## Introduction

Reduced muscle mass has been reported to be associated with health conditions such as diabetes (1), metabolic syndrome (2), and sarcopenia (3). Therefore, muscle mass gain (muscle hypertrophy) is important and of clinical significance. Muscle mass is regulated by maintaining a balance between muscle protein synthesis (MPS) and muscle protein breakdown. Resistance training (RT) is one of the effective ways to stimulate MPS (4) and leads to muscle hypertrophy (5). Considering that muscle mass in younger age groups has been reported to be associated with consequences such as sarcopenia (6, 7) or cardiovascular disease (8) in later life, the approach to muscle hypertrophy in young subjects is important.

Daily protein intake (PI) has been reported as the key factor for the regulation of muscle mass (9–19), and the pattern of daily PI in individuals is found to be typically lowest at breakfast and skewed toward dinner (13, 20, 21). Previous studies demonstrated that the typical pattern of daily PI was associated with lower muscle mass in the young population (13). A crossover study found that individuals having an evenly distributed PI over their daily meals had significantly greater 24-h MPS than those who had PI skewed toward evening meals, even though the diets were isoenergetic and isonitrogenous (22). In addition, Moore et al. (23) elucidated that adequate PI [0.24 g/kg body weight (BW)] is required to maximize MPS in the young population. Based on this threshold value, inadequate PI at breakfast is apparent in many of the previous studies (13, 21, 22). Furthermore, the habitual low PI at breakfast in the general population suggests that increased PI at breakfast can be an effective intervention to increase 24-h MPS and subsequently help achieve greater muscle mass during RT.

This study aimed to examine whether having an adequate PI at breakfast can be more effective in increasing muscle mass during a 12-wk RT program compared with typical PI pattern, skewed toward dinner.

## Methods

### Subjects

Thirty-three healthy young men (aged 18–26 y) were recruited verbally from June to July 2018 in this randomized clinical trial at Ritsumeikan University in Shiga, Japan. To be included, the participants needed to be nonsmokers who had not undergone any RT program for ≥1 y. Participants were excluded if they had any chronic diseases or were receiving any regular medication.

### Ethical approval

This study was approved by the Ethics Committee for Human Experiments at Ritsumeikan University (BKC-IRB-2018–017) and was conducted in accordance with the Declaration of Helsinki. This study was prospectively registered at https://www.umin.ac.jp/english/ as UMIN000037583. All 33 participants were informed about the experimental procedures, purpose of this study, and related risks and benefits before they provided written consent.

### Study design

This 12-wk intervention study required the random allocation of subjects to parallel groups. Random assignment of the subjects was conducted using sequences generated with a combination of RAND and RANK functions in Microsoft Excel by the primary investigator (JY). Group allocation was not discussed with the subjects. A total of 33 subjects were assigned to the following 2 groups: *1*) a “high breakfast” (HBR) group (*n* = 17) who consumed protein-enriched meals at breakfast to achieve >0.24 g/kg BW protein, which was reported to be the PI required at all 3 meals to maximize MPS (23); and *2*) a “low breakfast” (LBR) group (*n* = 16) who consumed a provided meal at breakfast to achieve a PI >0.24 g/kg BW at 2 meals (lunch and dinner). During the 12-wk trial, both groups continued the RT program 3 times per week. Subjects recorded their usual diets with dietary records at baseline and week 12 of the intervention. Strength assessment was conducted at baseline and week 12 to ensure the target working load [75–80% repetition maximum (RM)]. Anthropometric measurements were performed to evaluate body compositions at baseline and week 12.

### Dietary assessment

On the 3-d record, the subjects were allowed to note their dietary records on the usual meal days (13, 14, 18, 24). The dietary records included the following instructions: *1*) “Please note your dietary records on 2 weekdays and 1 weekend day”; *2*) “Please note all foods you had including confectionery or beverages”; *3*) “Please take pictures of foods or nutrition facts if it is cooked or processed food before you eat”; and *4*) “Please note your dietary records by referring to the examples provided.” Participants photographed the 3-d record using their phones to improve the accuracy of dietary assessment. Photographic data of the 3-d records were collected and confirmed by a registered dietitian via face-to-face interviews with the subjects. Before the commencement of the study, to ensure the accuracy of the dietary records, all subjects attended an explanatory meeting about the methodology of noting dietary records. The data were analyzed with Excel Eiyokun (version 8; Kenpakusha Co.) based on the Standard Table of Foods Composition in Japan 2015.

### Strength assessment

Maximum strength was assessed by 1-RM strength tests on the following weight-stack machines (all Life Fitness): leg curl, leg extension, arm curl, row, and chest press. Arm curl was conducted using the Dual Adjustable Pulley (Life Fitness) with a 20-in cable bar attachment and a preacher curl bench. A certified trainer evaluated 1-RM strength of the subjects at each test to ensure accuracy and safety. The 1-RM tests were based on the procedure recommended by the National Strength & Conditioning Association (25). On the 1-RM measurement day, participants completed a warm-up consisting of 1 set of 5–10 repetitions at a level of 40–60% of the estimated maximum. After a 1-min rest, the next practice was conducted, which involved 3–5 repetitions performed at a level 60–80% of the estimated maximum. After that, to determine 1-RM, 3–4 subsequent attempts were performed with progressively increasing weight until the participants failed. A 3-min rest was allowed between the 1-RM trials. The 1-RM tests were conducted at baseline and week 12 (2 d after the last training session of the RT program). None of the subjects experienced any joint pain and/or muscle soreness due to the 1-RM tests.

### RT program

The RT program was performed 3 times per week for 12 wk (36 sessions in total). Subjects could attend either the morning or afternoon sessions, and the researchers kept a record of the subjects’ sessions. The training consisted of a 5-repetition warm-up session on each machine with 50% of the 1-RM load, followed by 3 sets of 10 repetitions (the main RT program) on each machine until the subjects finished a familiarization period (sessions 1–8). After the familiarization period, subjects were asked to perform as many repetitions as they could in the third set, whereas the first and second sets were aimed at 10 repetitions. When the subjects had achieved >12 repetitions in the third set on a machine, the workload was increased by 5% of the former load on the machine for the next session. The performed workload differed as follows: 50% (sessions 1 and 2), 60% (sessions 3 and 4), 70% (sessions 5 and 6), 75% (sessions 7 and 8), and 75–80% (main RT program period, sessions 9–36). Resting periods of 2–3 min were allowed between sets. We confirmed the workload intensity based on the 1-RM tests at baseline and week 12. A certified trainer supervised all sessions.

### Dietary control

All subjects in both intervention groups were provided with standardized meals at breakfast for the entire intervention period. The provided meals consisted of 100 g yogurt (63.0 kcal, 3.60 g protein; Megumi, Megmilk Snow Brand Co.) and 50.0 g granola [Frugra: 220 kcal, 3.90 g protein; Choco Crunch & Banana: 224 kcal, 4.00 g protein; Mygra (no fruit): 225 kcal, 4.20 g protein; Walnut & Apple Maple: 226 kcal, 4.10 g protein; Tropical Coconut: 238 kcal, 4.00 g protein; and Orange Peel & Honey: 217 kcal, 4.00 g protein; all Calbee]. The subjects could choose 7 bags of granola from the 5 flavors. The energy and PI of the provided meals were based on the data of male subjects in our previous studies (283 kcal, 8.30 g protein) (13). All subjects completed a check sheet by selecting which flavor they had in the morning. The HBR group had the provided meal plus 1 serving of protein shakes (cocoa flavor: 83.0 kcal, 15.0 g protein; vanilla flavor: 82.0 kcal, 15.0 g protein; SAVAS WHEY PROTEIN 100, Meiji Holdings Co.) every morning, whereas the LBR group had the same shake with every dinner.

### Anthropometric measurements

The same radiological technician calibrated the DXA apparatus (Lunar Prodigy; GE Healthcare) at the beginning of each test day. After the calibration, lean soft tissue mass (LTM) and fat mass were analyzed with the subjects lying in the supine position. To standardize the DXA scan, the subjects arrived at the laboratory in the morning in fasting state on all measurement days. At week 12, the DXA measurement was performed within 1 wk of the last RT session. We used enCORE version 15 software (Lunar; GE Medical Systems), which generated automated measurements of LTM (arms, legs, and trunk) from total body scans. Subsequently, we calculated appendicular LTM (AppLTM) from measurements of the arms and legs. In addition, the precision of Lunar Prodigy DXA scan in repeated measurements was reported as ∼1.0% CV based on the results in previous studies (26–28).

### Statistical analysis

A power analysis based on previous research (29) showed that *n* = 13 was required for each group to detect between-group differences in total LTM using DXA when using a 2-sided statistical test (effect size = 1.50; α = 0.05; power = 0.95) (G*power version 3.1.9.2). Considering a possible dropout rate of 20% during the protocol, the final number of participants recruited was 16 per group.

All the data of group differences were analyzed using an independent *t* test. The dietary intake and body composition data were analyzed using a 2-factor repeated measures ANOVA to test interactions between time and condition. In cases of significant interactions, post hoc tests were performed with the independent *t* test for intergroup comparisons, and the paired *t* test for within-group comparisons. To examine the effects of the intervention on the main outcomes (body composition indices) without sample size, we calculated effect size (Cohen *d*), computed as the mean difference between the groups divided by the pooled SDs (30). The standard definitions of Cohen *d* are as follows: small, 0.30; medium, 0.50; large, 0.80; and very large, 1.30 (30, 31).

All statistical analyses were performed using SPSS version 23.0 for Windows (IBM Corp.). *P* values <0.05 using 2-tailed tests were considered statistically significant. All data are expressed as means ± SEs.

## Results

### Subject participation

**FIGURE 1:**
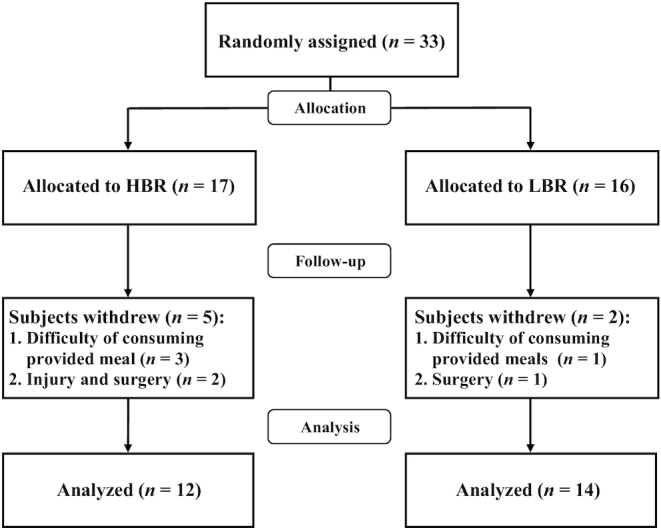
Flow diagram of the 12-wk clinical trial in healthy young men with high or low relative protein intakes at breakfast. The HBR (“high breakfast”) group consumed a protein-enriched meal at breakfast to achieve a protein intake >0.24 g/kg body weight, reported as the required protein intake at all 3 meals to maximize muscle protein synthesis (23). The LBR (“low breakfast”) group consumed a provided meal at breakfast to achieve a protein intake >0.24 g/kg body weight at only 2 meals (lunch and dinner).

Although we recruited 33 healthy young men (HBR *n* = 17; LBR *n* = 16), a total of 26 subjects (HBR *n* = 12; LBR *n* = 14) were included in the final analysis because 5 from the HBR group and 2 from the LBR group were excluded due to various reasons (**Figure 1**). Although there was no harmful effect of the provided meals, 3 subjects in the HBR group and 1 in the LBR group occasionally could not consume the provided meals during the intervention period. In addition, 2 subjects in the HBR group and 1 in the LBR group were excluded because of injuries and surgical treatments, which were not related to the intervention in the present study. A total of 26 subjects completed all RT sessions without any injuries.

### Nutrient intakes

We confirmed that 16/26 subjects (61.5%) at breakfast, 4 (15.4%) at lunch, and 1 (3.8%) at dinner did not achieve a PI of 0.24 g/kg BW at baseline. We considered the compliance of consuming provided meals to be reliable based on the check sheets, which showed >95% compliance in both groups (HBR group: 96.7 ± 0.9%; LBR group: 95.7 ± 0.9%) for the entire period. The daily energy intakes and absolute and relative macronutrient intakes at baseline did not differ between the groups (*P* = 0.133–0.971) (**Table 1**).

**FIGURE 2:**
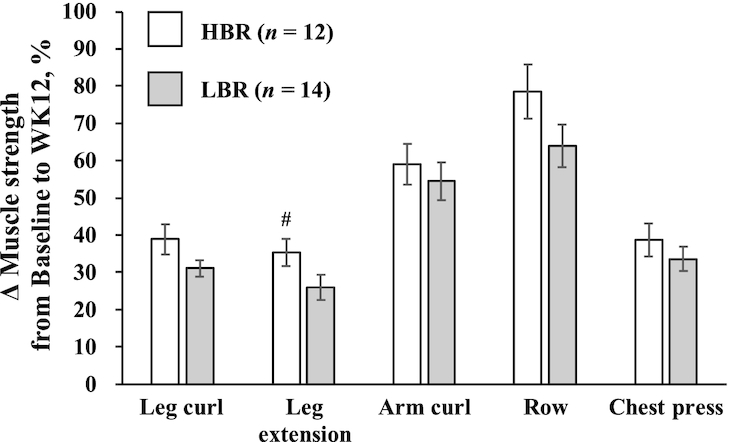
Comparison of percentage change in muscle strength from baseline to week 12 (WK12) in healthy young men with high (HBR) or low (LBR) relative protein intakes at breakfast. Values are indicated as means ± SEs, *n* = 12 (HBR) and *n* = 14 (LBR). Statistical analysis was performed with an independent *t* test to compare percentage change from baseline to week 12 in muscle strength between groups. ^#^Tended to differ from LBR, *P *= 0.069. The HBR (“high breakfast”) group consumed a protein-enriched meal at breakfast to achieve a protein intake >0.24 g/kg body weight, reported as the required protein intake at all 3 meals to maximize muscle protein synthesis (23). The LBR (“low breakfast”) group consumed a provided meal at breakfast to achieve a protein intake >0.24 g/kg body weight at only 2 meals (lunch and dinner).

**FIGURE 3:**
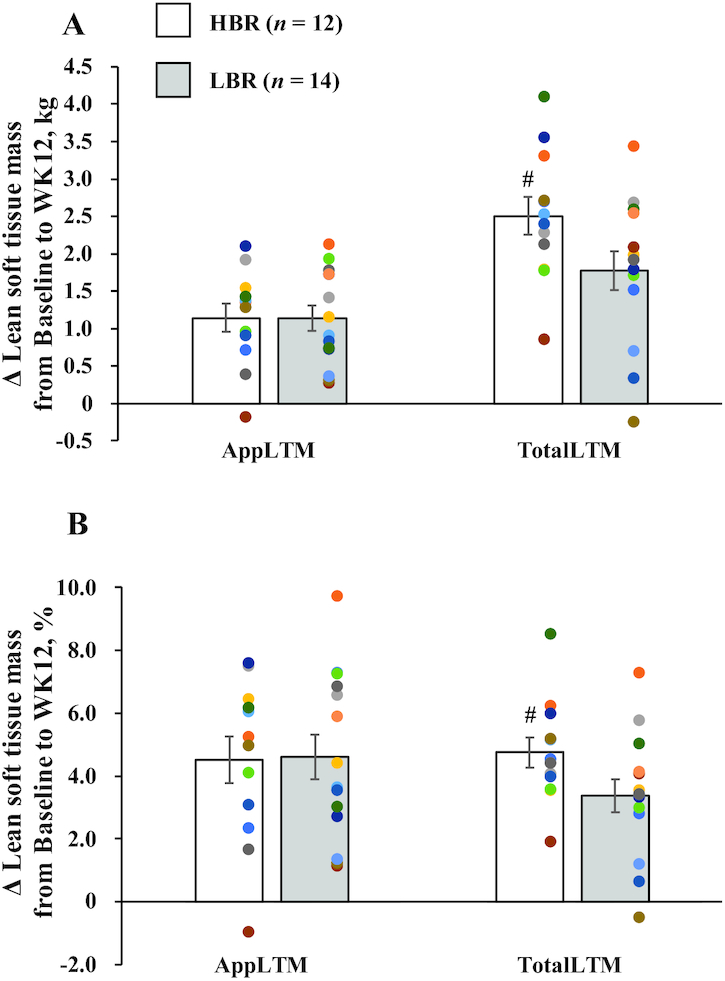
Comparison of absolute change (A) and percentage change (B) in AppLTM and TotalLTM from baseline to week 12 in healthy young men with high or low relative protein intakes at breakfast. Values are indicated as means ± SEs, *n* = 12 (HBR) and *n* = 14 (LBR). Statistical analysis was performed with an independent *t* test to compare absolute and percentage changes from baseline to week 12 in AppLTM and TotalLTM between groups. Cohen *d* was used to express the effect size of comparisons (standard definitions: small, 0.3; medium, 0.5; large, 0.8; very large, 1.30). ^#^Tended to differ from LBR, *P* = 0.056 (A: *d* = 0.795) or 0.067 (B: *d* = 0.760). The HBR (“high breakfast”) group consumed a protein-enriched meal at breakfast to achieve a protein intake >0.24 g/kg body weight, reported as the required protein intake at all 3 meals to maximize muscle protein synthesis (23). The LBR (“low breakfast”) group consumed a provided meal at breakfast to achieve a protein intake >0.24 g/kg body weight at only 2 meals (lunch and dinner). AppLTM, appendicular lean soft tissue mass; TotalLTM, total lean soft tissue mass; WK12, week 12.

**TABLE 1:** Daily energy and nutrient intake at baseline and week 12 in healthy young men with high or low relative protein intakes at breakfast^1^

	HBR (*n* = 12)		LBR (*n* = 14)		Repeated measures ANOVA		
	Baseline	Week 12	Baseline	Week 12	Group	Time	Group × time
Total							
Energy, kcal	2599 ± 132	2456 ± 147	2453 ± 106	2543 ± 114	0.837	0.799	0.274
Protein, g	80.9 ± 4.63	89.4 ± 5.51	83.1 ± 4.91	97.1 ± 3.46	0.384	0.004	0.444
Protein, g/kg BW	1.23 ± 0.08	1.30 ± 0.07	1.27 ± 0.07	1.45 ± 0.04	0.234	0.019	0.314
Fat, g	80.9 ± 6.91	84.4 ± 8.22	74.7 ± 4.48	94.5 ± 5.55	0.784	0.049	0.158
Carbohydrate, g	374 ± 20.2	323 ± 16.4	343 ± 16.8	315 ± 15.1	0.331	0.014	0.433
Breakfast							
Energy, kcal	508 ± 82.0	374 ± 2.00	434 ± 73.8	287 ± 0.89	0.157	0.018	0.909
Protein, g	15.5 ± 2.30	22.6 ± 0.02^†^	13.6 ± 2.64	7.68 ± 0.03^*†^	<0.001	0.729	0.001
Protein, g/kg BW	0.23 ± 0.04	0.33 ± 0.01^†^	0.21 ± 0.04	0.12 ± 0.00^*†^	0.001	0.976	0.002
Fat, g	16.2 ± 3.29	13.5 ± 0.37	14.1 ± 2.72	11.6 ± 0.16	0.346	0.251	0.976
Carbohydrate, g	74.1 ± 13.0	42.5 ± 0.37	62.2 ± 10.5	40.4 ± 0.15	0.405	0.003	0.557
Lunch							
Energy, kcal	730 ± 57.8	921 ± 53.2	796 ± 43.2	943 ± 56.2	0.422	0.003	0.674
Protein, g	21.7 ± 2.54	31.8 ± 2.68	26.9 ± 2.18	30.0 ± 1.71	0.535	0.001	0.056
Protein, g/kg BW	0.33 ± 0.04	0.46 ± 0.03	0.41 ± 0.03	0.45 ± 0.03	0.354	0.003	0.090
Fat, g	21.2 ± 2.42	31.1 ± 3.42	20.0 ± 2.42	34.4 ± 2.95	0.728	<0.001	0.415
Carbohydrate, g	110 ± 8.70	122 ± 7.56	123 ± 6.33	124 ± 7.61	0.274	0.423	0.505
Dinner							
Energy, kcal	1144 ± 72.5	1038 ± 98.4	1121 ± 75.5	1147 ± 82.2	0.656	0.561	0.339
Protein, g	39.7 ± 3.59	32.4 ± 3.06	40.8 ± 3.70	55.4 ± 3.17^*†^	0.007	0.184	<0.001
Protein, g/kg BW	0.60 ± 0.06	0.48 ± 0.05	0.62 ± 0.05	0.83 ± 0.04^*†^	0.002	0.326	<0.001
Fat, g	36.7 ± 5.50	36.8 ± 5.72	36.5 ± 4.27	43.1 ± 5.21	0.607	0.458	0.469
Carbohydrate, g	155 ± 6.78	135 ± 11.9	143 ± 9.69	126 ± 8.97	0.367	0.025	0.875

1 Values are means ± SEs. *Different from HBR at that time; ^†^different from baseline within a group; *P* < 0.05. BW, body weight; HBR, “high breakfast”—consuming a protein-enriched meal at breakfast to achieve a protein intake >0.24 g/kg BW at all 3 meals; LBR, “low breakfast”—consuming a provided meal at breakfast to achieve a protein intake >0.24 g/kg BW at 2 meals (lunch and dinner).

During the intervention, there were significant time × treatment interactions for absolute and relative PIs, being higher at breakfast and lower at dinner in the HBR than in the LBR group at week 12 (Table 1) (*P *< 0.001). The HBR group had 0.33 ± 0.01 g protein/kg BW at breakfast, 0.46 ± 0.03 at lunch, 0.48 ± 0.05 at dinner, and 1.30 ± 0.07 in total, whereas PIs in the LBR group were 0.12 ± 0.00 at breakfast, 0.45 ± 0.03 at lunch, 0.83 ± 0.04 at dinner, and 1.45 ± 0.04 in total. In addition, the post hoc tests showed that absolute PIs (*P* = 0.010) and relative PIs (*P* = 0.015) at breakfast in the HBR group significantly increased throughout the intervention. Similarly, compared with baseline, absolute and relative PIs (both *P* < 0.001) at dinner in the LBR group significantly increased whereas absolute PIs (*P* = 0.044) and relative PIs (*P* = 0.039) at breakfast significantly decreased in the LBR group at week 12. The total intakes of protein, fat, and carbohydrate; carbohydrate intake at breakfast; energy and fat intakes at lunch; and carbohydrate intake at dinner, also changed over time (*P* < 0.05) (Table 1).

### RT program and strength assessment

There were no significant differences in 1-RM values on all machines between the 2 groups at baseline [total (*P* = 0.236), leg curl (*P* = 0.221), leg extension (*P* = 0.090), arm curl (*P* = 0.601), row (*P* = 0.941), and chest press (*P* = 0.447)], and no significant group × time interaction was observed on each machine from baseline to week 12 (**Table 2**). The main effects of time on each 1-RM value were observed, and the RT program significantly increased the 1-RM values on each machine. We found no significant differences in percentage change in 1-RM values on each machine (**Figure 2**), although the percentage change of leg extension in the HBR group tended to be greater than that in the LBR group (HBR compared with LBR: 35.2 ± 3.59% compared with 25.9 ± 3.34%; *P* = 0.069). There were no significant differences in total workload (HBR compared with LBR: 32.8 ± 1.36 × 10^4^ kg compared with 33.1 ± 0.90 × 10^4^ kg; *P*  = 0.829) and repetitions (HBR compared with LBR: 53.8 ± 0.48 × 10^2^ reps compared with 53.6 ± 0.29 × 10^2^ reps; *P* = 0.702) for the entire training period between the 2 groups, whereas the HBR group attended significantly fewer morning exercise sessions than the LBR group (HBR compared with LBR: 37.3 ± 5.2% compared with 57.1 ± 5.3%; *P* = 0.014).

**TABLE 2:** 1-RM values at baseline and week 12 in healthy young men with high or low relative protein intakes at breakfast^1^

	HBR (*n* = 12)		LBR (*n* = 14)		Repeated measures ANOVA		
	Baseline	Week 12	Baseline	Week 12	Group	Time	Group × time
Total, kg	326 ± 11.1	469 ± 17.9	349 ± 11.1	470 ± 13.0	0.510	<0.001	0.109
Leg curl, kg	92.5 ± 3.00	128 ± 4.71	97.8 ± 2.61	128 ± 4.59	0.592	<0.001	0.240
Leg extension, kg	131 ± 4.45	176 ± 6.20	142 ± 4.41	178 ± 4.61	0.334	<0.001	0.096
Arm curl, kg	28.2 ± 1.27	44.9 ± 2.64	29.1 ± 1.29	44.4 ± 1.53	0.922	<0.001	0.531
Row, kg	44.2 ± 2.45	78.1 ± 4.23	44.4 ± 2.04	72.0 ± 2.89	0.454	<0.001	0.094
Chest press, kg	30.8 ± 2.18	42.1 ± 2.29	33.5 ± 2.15	44.0 ± 2.36	0.478	<0.001	0.603

1 Values are means ± SEs. HBR, “high breakfast”—consuming a protein-enriched meal at breakfast to achieve a protein intake >0.24 g/kg BW at all 3 meals; LBR, “low breakfast”—consuming a provided meal at breakfast to achieve a protein intake >0.24 g/kg BW at 2 meals (lunch and dinner); 1-RM, 1-repetition maximum.

### Anthropometric status

The height, BW, BMI, and any body composition data at baseline did not differ between the groups (*P* = 0.478–0.936) (**Table 3**). Although there were no significant interactions, the main effects of time on BW, BMI, AppLTM, total LTM, and body fat percentage were observed. The RT program significantly increased BW, BMI, AppLTM, and total LTM from baseline to week 12, whereas body fat percentage was significantly decreased. We also determined the absolute changes and percentage changes in AppLTM and total LTM (**Figure 3**A, B). The HBR group had greater increase in total LTM (HBR compared with LBR: 2.50 ± 0.25 kg compared with 1.77 ± 0.26 kg; *P* = 0.056; *d* = 0.795) than the LBR group, although there was no significant difference in the change of AppLTM (HBR compared with LBR: 1.14 ± 0.18 kg compared with 1.14 ± 0.17 kg; *P* = 0.991; *d* = 0.004; Figure 3A). We observed similar results in the percentage changes of AppLTM (HBR compared with LBR: 4.52 ± 0.74% compared with 4.62 ± 0.71%; *P* = 0.924; *d* = 0.038) and total LTM (HBR compared with LBR: 4.76 ± 0.48% compared with 3.36 ± 0.53%; *P* = 0.067; *d* = 0.760; Figure 3B).

**TABLE 3:** Anthropometric status at baseline and week 12 in healthy young men with high or low relative protein intakes at breakfast^1^

	HBR (*n* = 12)		LBR (*n* = 14)		Repeated measures ANOVA		
	Baseline	Week 12	Baseline	Week 12	Group	Time	Group × time
Age, y	20.3 ± 0.51		21.2 ± 0.54				
Height, m	1.73 ± 0.01	1.73 ± 0.01	1.72 ± 0.01	1.72 ± 0.01	0.542	0.563	0.317
BW, kg	66.8 ± 1.96	68.8 ± 2.02	65.3 ± 1.82	67.1 ± 1.87	0.568	<0.001	0.702
BMI, kg/m^2^	22.2 ± 0.60	22.9 ± 0.58	22.1 ± 0.55	22.7 ± 0.53	0.862	<0.001	0.479
AppLTM, kg	25.0 ± 0.69	26.2 ± 0.74	24.9 ± 0.64	26.0 ± 0.69	0.881	<0.001	0.991
TotalLTM, kg	52.4 ± 1.32	54.9 ± 1.37	53.4 ± 1.22	55.1 ± 1.27	0.752	<0.001	0.056
Bone mineral content, kg	3.06 ± 0.09	3.07 ± 0.09	3.04 ± 0.08	3.06 ± 0.08	0.889	0.084	0.843
Body fat, kg	11.3 ± 1.31	10.8 ± 1.37	8.91 ± 1.22	8.94 ± 1.27	0.254	0.273	0.214

1 Values are means ± SEs. AppLTM, appendicular lean soft tissue mass; BW, body weight; HBR, “high breakfast”—consuming a protein-enriched meal at breakfast to achieve a protein intake >0.24 g/kg BW at all 3 meals; LBR, “low breakfast”—consuming a provided meal at breakfast to achieve a protein intake >0.24 g/kg BW at 2 meals (lunch and dinner); TotalLTM, total lean soft tissue mass.

## Discussion

We conducted a 12-wk randomized controlled, parallel-group clinical trial to examine the effectiveness of protein-enriched meals at breakfast for RT-induced muscle hypertrophy in healthy young men. To our knowledge, this is the first study to describe that having adequate PI (>0.24 g/kg BW) at all 3 meals by adding a high-protein breakfast was more effective in increasing muscle mass in healthy young participants than a typical PI pattern (skewed PI toward dinner), while maintaining a daily total PI that was not significantly different between the 2 groups (*P* = 0.236). We observed greater total LTM gain in the HBR group than in the LBR group, but there was no significant difference in AppLTM gain between groups; this type of differential response between total and appendicular LTM has been reported in other studies (32–34). Because our resistance exercise protocol focused on training major muscle groups in both the lower and upper body (using chest press and row), change in trunk musculature can only be assessed with total LTM (which also includes organ tissues). Therefore, we believe that the total LTM assessment can still reflect muscle hypertrophy with our training protocol.

To date, total daily PI is considered one of the most important factors for the regulation of muscle mass based on several epidemiological studies (9–13) and intervention studies (14–16, 19). However, dietary PI over 3 meals is reported to be skewed toward dinner, being lowest at breakfast in people in Japan (13, 20) and the United States (21). Our previous study elucidated the skewness of daily PI patterns and lack of PI at breakfast (0.20 g/kg BW) in college and graduate students (13) according to the threshold value (0.24 g/kg BW) in the previous study by Moore et al (23). Likewise in the present study, dietary PI was lowest at breakfast and highest at dinner at baseline. Our previous cross-sectional study reported that the skewed PI pattern caused by the inadequate PI at breakfast was associated with the risk of developing reduced muscle mass (13). In respect of nutritional interventions, Moore et al. (23) evaluated MPS in healthy young subjects in response to various amounts (0–40 g) of high-quality protein as a single bolus using stable isotope methodology. Results from this study identified 0.24 g protein/kg BW as the amount of protein required to maximize MPS at a single protein consumption. In other words, inadequate protein at meals can negatively affect stimulation of MPS throughout a day. Thus, well-reported dietary habits such as skipping breakfast (35), having inadequate protein at breakfast, and consuming too much protein at dinner, which was observed in the present study, can be risk factors for reduced muscle mass (13, 18). Another crossover study investigated effects on 24-h MPS of evenly distributed PI over breakfast, lunch, and dinner (EVEN: PI = 0.41, 0.39, and 0.43 g/kg BW, respectively) or skewed PI (SKEW; PI = 0.14, 0.21, and 0.83 g/kg BW, respectively) for 7 d (22). This study showed that 24-h MPS at both days 1 and 7 in the EVEN group was significantly higher than that in the SKEW group, even though the total energy and PI did not differ between the groups. Results from this study emphasize that inadequate PI at breakfast and lunch, as in the SKEW group, adversely affects the stimulation of MPS, regardless of the total daily PI. During the intervention in the present study, the HBR group had an adequate PI over 3 meals, whereas the LBR group had an inadequate PI at breakfast. In addition, both groups had total daily PI above the Japanese RDA level (0.9 g/kg BW), and there was no significant difference in the total daily PI between groups. Generally, having inadequate protein at meals, especially at breakfast, negatively affects muscle hypertrophy through the RT program. However, further studies focusing on the relation between PI at each meal and the regulation of muscle mass, especially with an exercise program, are needed.

There are several limitations to the present study. Firstly, only 12 of the 17 subjects in the HBR group completed the 12-wk intervention, when our power analysis required 13 subjects in each group. Thus, we calculated Cohen *d* indicating the effect size of intervention without the sample size (30). The effect size with Cohen *d* indicated considerable effects in the between-group comparison of the change in total LTM, which supports the validity of our results. Secondly, we did not designate a specific training time; subjects were free to undergo RT sessions either in the morning or in the afternoon. However, systematic reviews with meta-analysis revealed that there were no further benefits of PI timing (36) or RT timing (37) on training-induced muscle hypertrophy. Thirdly, although muscle hypertrophy has been reported to be positively correlated with muscle strength (38), we did not detect a statistically significant impact of the intervention on muscle strength. However, 1-RM assessment using specific training machines for both groups is a robust method, and might not be adequately sensitive for detecting the small but significant difference in muscle gain between groups. A further study assessing isometric and/or isokinetic strength is required to clarify the discrepancy between the group differences in LTM, and the absence of a group difference in strength. Finally, the dietary nutrient intake data are dependent on self-reported dietary records, which are said to suffer from underreporting (39). However, dietary records are reportedly a more precise tool than FFQs (40).

In conclusion, the present study indicates that achieving adequate PI (≥0.24 g/kg BW) at breakfast and subsequent meals induces higher RT-induced muscle hypertrophy compared with a skewed PI pattern with insufficient PI at breakfast. To maximize muscle accretion with RT, not only daily total PI but also PI at each meal, especially at breakfast, should be considered.

## Notes

This work was supported by the Japan Society for the Promotion of Science, Grants-in-Aid for Scientific Research (no. 17H02183 to SF).

Author disclosures: The authors report no conflicts of interest.

The corresponding author will cooperate with any requests from the journal for data or additional information should questions about the article arise after publication.

Abbreviations used: AppLTM, appendicular lean soft tissue mass; BW, body weight; HBR, “high breakfast”—consuming a protein-enriched meal at breakfast to achieve a protein intake >0.24 g/kg BW at all 3 meals; LBR, “low breakfast”—consuming a provided meal at breakfast to achieve a protein intake >0.24 g/kg BW at 2 meals; LTM, lean soft tissue mass; MPS, muscle protein synthesis; PI, protein intake; RT, resistance training; 1-RM, 1-repetition maximum.
